# Depression, Cognitive Impairment and Education Over the Life Course: Comparing China and the US

**DOI:** 10.1093/geroni/igaf122.560

**Published:** 2025-12-31

**Authors:** Nan Zhou

**Affiliations:** Case Western Reserve University, Cleveland, Ohio, United States

**Keywords:** Educational attainment, depression, cognitive aging, cross-national comparison, Cumulative Inequality Theory

## Abstract

Depression and cognitive impairment frequently co-occur in later life, yet their longitudinal relationship—and how it varies by educational attainment and national context—remains underexplored. This study examines the bidirectional association between depressive symptoms and cognitive impairment among older adults in China and the United States, assessing differences across education levels. Using longitudinal data from 2011 to 2020 from the China Health and Retirement Longitudinal Study (CHARLS) and the U.S. Health and Retirement Study (HRS), this study employs a multi-group cross-lagged panel model (CLPM) to examine the relationship of depressive symptoms and cognitive impairment, its stability over the life course and its relation to education. Measurement and structural invariance across four educational attainment groups: less than high school, high school diploma, associate’s degree, and bachelor’s degree or higher. The study explores the hypotheses that 1) depression and cognitive impairment have a bidirectional relationship within each educational group, 2) individuals with lower educational attainment will exhibit stronger reciprocal effects between depression and cognitive impairment, and 3) that the association may be more pronounced in China, where lower education levels may be linked to steeper cognitive decline, while higher education mat serve as a protective factor. Differences in healthcare access, social policies, and cultural attitudes toward aging may further shape these cross-national patterns. Findings may contribute to understanding how education influences later-life mental health trajectories and provide insights for policies and interventions aimed at reducing disparities in cognitive aging.

